# The USDA-ARS Ag100Pest Initiative: High-Quality Genome Assemblies for Agricultural Pest Arthropod Research

**DOI:** 10.3390/insects12070626

**Published:** 2021-07-09

**Authors:** Anna K. Childers, Scott M. Geib, Sheina B. Sim, Monica F. Poelchau, Brad S. Coates, Tyler J. Simmonds, Erin D. Scully, Timothy P. L. Smith, Christopher P. Childers, Renee L. Corpuz, Kevin Hackett, Brian Scheffler

**Affiliations:** 1 Bee Research Laboratory, Beltsville Agricultural Research Center, Agricultural Research Service, USDA, 10300 Baltimore Avenue, Beltsville, MD 20705, USA; 2 Tropical Crop and Commodity Protection Research Unit, Daniel K Inouye U.S. Pacific Basin Agricultural Research Center, Agricultural Research Service, USDA, 64 Nowelo Street, Hilo, HI 96720, USA; scott.geib@usda.gov (S.M.G.); sheina.sim@usda.gov (S.B.S.); tyler.simmonds@usda.gov (T.J.S.); renee.corpuz@usda.gov (R.L.C.); 3 National Agricultural Library, Agricultural Research Service, USDA, 10301 Baltimore Avenue, Beltsville, MD 20705, USA; monica.poelchau@usda.gov (M.F.P.); christopher.childers2@usda.gov (C.P.C.); 4 Corn Insects & Crop Genetics Research Unit, Agricultural Research Service, USDA, 2310 Pammel Dr., Ames, IA 50011, USA; brad.coates@usda.gov; 5 Oak Ridge Institute for Science and Education, P.O. Box 117, Oak Ridge, TN 37831, USA; 6 Stored Product Insect and Engineering Research Unit, Center for Grain and Animal Health Research, Agricultural Research Service, USDA, 1515 College Avenue, Manhattan, KS 66502, USA; erin.scully@usda.gov; 7 Genetics and Breeding Research Unit, U.S. Meat Animal Research Center, Agricultural Research Service, USDA, State Spur 18D, Clay Center, NE 68933, USA; tim.smith2@usda.gov; 8 Office of National Programs, Crop Production and Protection, Agricultural Research Service, USDA, 5601 Sunnyside Avenue, Beltsville, MD 20705, USA; kevin.hackett@usda.gov; 9 Genomics and Bioinformatics Research Unit, Jamie Whitten Delta States Research Center, Agricultural Research Service, USDA, 141 Experiment Station Road, Stoneville, MS 38776, USA; brian.scheffler@usda.gov

**Keywords:** Arthropoda, pests, invasive pests, genome sequencing, long-read sequencing, low-input DNA, HiC scaffolding, genome assembly, genomics

## Abstract

**Simple Summary:**

High-quality genome assemblies are essential tools for modern biological research. In the past, creating genome assemblies was prohibitively expensive and time-consuming for most non-model insect species due to, in part, the technical challenge of isolating the necessary quantity and quality of DNA from many species. Sequencing methods have now improved such that many insect genomes can be sequenced and assembled at scale. We created the Ag100Pest Initiative to propel agricultural research forward by assembling reference-quality genomes of important arthropod pest species. Here, we describe the Ag100Pest Initiative’s processes and experimental procedures. We show that the Ag100Pest Initiative will greatly expand the diversity of publicly available arthropod genome assemblies. We also demonstrate the high quality of preliminary contig assemblies. We share arthropod-specific technical details and insights that we have gained during the project. The methods and preliminary results presented herein should help other researchers attain similarly high-quality assemblies, effectively changing the landscape of insect genomics.

**Abstract:**

The phylum Arthropoda includes species crucial for ecosystem stability, soil health, crop production, and others that present obstacles to crop and animal agriculture. The United States Department of Agriculture’s Agricultural Research Service initiated the Ag100Pest Initiative to generate reference genome assemblies of arthropods that are (or may become) pests to agricultural production and global food security. We describe the project goals, process, status, and future. The first three years of the project were focused on species selection, specimen collection, and the construction of lab and bioinformatics pipelines for the efficient production of assemblies at scale. Contig-level assemblies of 47 species are presented, all of which were generated from single specimens. Lessons learned and optimizations leading to the current pipeline are discussed. The project name implies a target of 100 species, but the efficiencies gained during the project have supported an expansion of the original goal and a total of 158 species are currently in the pipeline. We anticipate that the processes described in the paper will help other arthropod research groups or other consortia considering genome assembly at scale.

## 1. Introduction

Agricultural pest arthropods damage crops and endanger animal and human health both directly through disease and indirectly by threatening global food supply. Specifically, herbivorous and parasitic insects impact plant and animal health, respectively, through direct feeding or by vectoring disease-causing viruses and pathogens. In the case of zoonotic diseases, the impacts on humans are compounded with effects on animal food production and human health. For example, ticks and tick-borne pathogens pose a major threat to US public health and livestock production, with the economic damage for Lyme disease alone estimated at up to USD 4.8–9.6 billion per year [1]. Herbivorous insects can dramatically reduce the quantity and quality of products both pre- and post-harvest. An estimated 6% of maize production is lost to insect pests in the United States annually [2], which is over USD 3 billion annually using the latest production data [3] and a corn market price of USD 3.75 per bushel. The western corn rootworm (*Diabrotica virgifera virgifera*) alone was responsible for USD 1.4 billion in direct production losses in 2010 [4].

One grand challenge facing agriculture is the need to increase production by up to 70% to meet the demands of a human population anticipated to reach 10 billion by 2050 [5] while simultaneously reducing environmental impacts and meeting the challenges posed by climate change. The threats to agriculture by insects are pernicious and ever-increasing, and pest control presents major hurdles for achieving 2050 production needs [6]. Insects are not a new threat to agriculture, but their impacts on production have been greatly affected by pesticide use, climate change, and the introduction of non-native insects into new habitats and landscapes through the shipping of infested materials and agricultural products around the globe. Widespread insecticide resistance among arthropod pest species has emerged [7,8], expanded seasonal activity and geographic ranges of native pests have increased damage [9], and the migration of non-native pests between habitats has challenged ecosystems [10]. Our ability to control arthropod pests must undoubtedly also evolve and adapt to mitigate these threats, and genomics, in particular, holds promise to facilitate the development of innovative and resilient control technologies.

Genome assemblies provide comprehensive information about the genome that cannot be matched by transcriptome sequencing and assembly. Full genome assemblies are not restricted to a subset of expressed regions that can easily miss gene duplications, regulatory components, and genes with low expression levels. Non-transcribed regions of the genome can influence gene expression in various ways [11,12,13,14,15]. For example, promoters, enhancers, and other DNA segments more commonly impact gene regulation compared to protein-coding regions of the genome, which can have strong impacts on phenotype [16,17,18,19]. In addition, non-translated RNAs, such as microRNAs or long non-coding RNAs (lncRNAs) that are not identified in typical transcriptome sequencing, can play key roles in establishing phenotypes and improve our understanding of how insects interact with their plant hosts and adapt to changing environmental conditions [20,21]. Recent estimates suggest that nearly 90% of economically or ecologically important traits in organisms may be determined by variation in non-coding regions of the genome [22], indicating the need for high-quality reference genome assemblies to study traits relevant to pest management.

Large-scale genome sequencing initiatives such as i5k, the initiative committed to sequencing 5000 arthropod genomes [23,24], are developing the infrastructure to build reference-quality genome assemblies to facilitate basic and applied research that will lead to improved pest management tactics. A pilot project of the i5k produced genome assemblies of 28 species and greatly improved our understanding of the challenges of sequencing arthropods [25]. More recently, the Earth BioGenome Project (EBP) has brought together numerous affiliated consortia to produce reference-quality genome assemblies from species across the tree of life, with the ultimate goal of sequencing all eukaryotes over a 10-year period [26]. The Ag100Pest Initiative [27] is a bold endeavor by the United States Department of Agriculture, Agricultural Research Service (USDA-ARS) to generate reference-quality genome assemblies for the top 100 US agricultural pest arthropod species, thus advancing the missions of both the i5k Initiative and the EBP [26].

**Figure 1:**
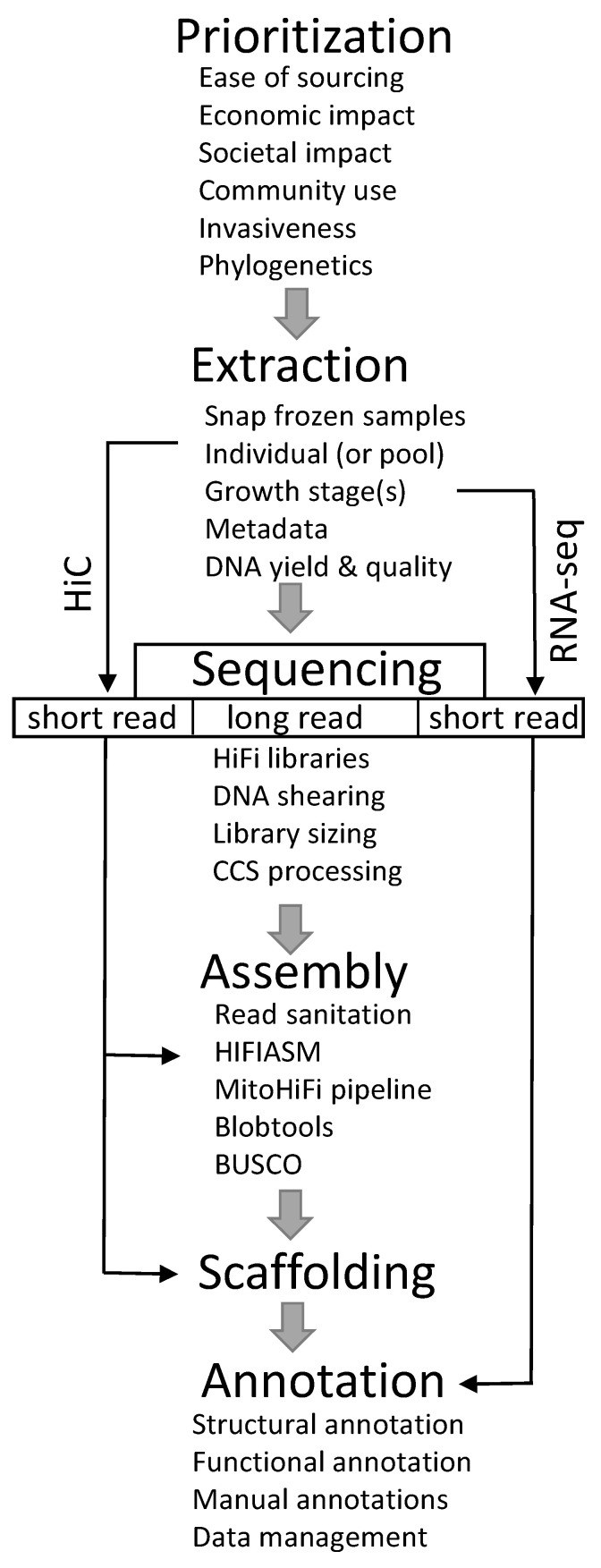
Workflow used by Ag100Pest to generate annotated reference-quality assemblies.

The USDA-ARS performs research to support the health of beneficial arthropods and control the damaging effects of pests in order to enhance food security and human health [28,29]. This article describes the framework for the Ag100Pest Initiative, encompassing the scope, operation, and challenges and lessons learned since inception. The Ag100Pest Initiative is developing low-cost, high-quality reference genomes from single insect specimens, including insects of large and small physical and genome size. Organizing a coordinated initiative to address these goals is not a trivial undertaking; it requires adequate infrastructure, streamlined and effective methodologies for library production, sequencing and bioinformatic analysis, operational and administrative schemata, and, of course, funding. Technological aspects will undoubtedly change as sequencing and assembly methods evolve, but the Ag100Pest Initiative framework and operational advances can inform those currently involved in or planning analogous endeavors. Ag100Pest has developed a pipeline using a combination of long-read sequencing from a single specimen and HiC scaffolding, along with companion RNA expression data, to generate annotated genome assemblies that meet or exceed EBP standards (Figure 1). This effort is greatly changing the landscape of insect genomics research, and we hope that by sharing our insights, others will join in this revolution.

## 2. Materials and Methods

### 2.1. Species Prioritization

Ag100Pest consulted several external groups in the process of species selection, including the USDA Animal and Plant Health Inspection Service (USDA-APHIS), the Federal Interagency Committee on Invasive Terrestrial Animals and Pathogens [30], the Cooperative Agricultural Pest Survey [31], and the broader arthropod research community as well as USDA researchers. A diverse set of pest species nominations, including those with economically significant effects on field crops, animals, bees, forests, and stored products, were sought from across agricultural stakeholders. Several factors were taken into consideration (Figure 1), and species with strong supporting research communities were prioritized. Although the focus is on agricultural pests in the United States, we also included pests with the potential to become established invasive species or those of international importance.

### 2.2. Sample Collection and Extraction

Samples for sequencing are collected fresh, snap-frozen in liquid nitrogen, and shipped on dry ice when feasible (Figure 1). Relevant metadata information is cataloged according to the NCBI Invertebrate 1.0 metadata format [32]. Once received and queued, whole single insect specimens are assessed for the feasibility of generating both Pacific Biosciences (PacBio) High-Fidelity (HiFi) libraries and HiC libraries from the same specimen (Figure 1). When single individual specimens are too small to generate both libraries from the same specimen, one individual is used for HiFi library preparation, and a separate specimen or pool of individuals is used for HiC. DNA extraction is performed to optimize yield and fragment size (≥50 kb). Compared with PacBio continuous long-read (CLR) libraries or those for Oxford Nanopore, there is no advantage of having ultra-high molecular weight DNA (at the megabase scale) for HiFi libraries. This aspect simplifies the DNA extraction step, where the yield and integrity of extracted DNA are the focus. 

DNA extraction begins by grinding the tissue into a powder using cryogenically chilled aluminum blocks and a SPEX GenoGrinder (SPEX SamplePrep LLC, Metuchen, NJ, USA). This powder is used for input into the MagAttract high molecular weight (HMW) DNA extraction kit (Qiagen, Hilden, Germany), where the lysis steps are scaled to the size of the insect. After extraction, DNA integrity is determined by capillary electrophoresis on an Agilent fragment analyzer or Femtopulse (Agilent Technologies, Santa Clara, CA, USA) to determine fragment size range. Spectrophotometric (e.g., absorbance at 230, 260, and 280 nm) and fluorometric (EvaGreen/Qubit) methods are used to estimate purity and quantity, respectively. 

### 2.3. Library Preparation, Sequencing, and Assembly

Prior to library preparation, a minimum input of 300 ng of DNA is sheared to the target fragment length between 10 and 20 kb using a Diagenode Megaruptor (Diagenode Inc., Denville, NJ, USA). This sheared DNA is processed for HiFi library construction using the SMRTBell Express Template Prep Kit 2.0 with the optional Enzyme Clean Up Kit 2.0 (Pacific Biosciences, Menlo Park, CA, USA), but higher or lower input may be required based on the quality of the DNA, the amount of data needed (and, thus, number of SMRTcells to be sequenced), and the method of final size selection of the library. Stringent size selection is typically not performed on the final library; rather, a modified AMPure cleanup step is used to remove library fragments < 3 kb. More stringent sizing is typically only performed if the library has a large number of fragments smaller than 8 kb or if the library concentration is sufficient to allow sizing on a BluePippin (as a high-pass) or SageELF (as a fraction or set of fractions; Sage Science Inc., Beverly, MA, USA) and still retain sufficient library volume for loading. Sequence data is collected on a PacBio Sequel II system and processed through circular consensus sequencing (CCS) to generate ~99.9% accurate, single-molecule High-Fidelity (HiFi) reads [33]. In our process (Figure 1), the HiFi reads are then pre-processed to remove any PacBio adapter contamination [34] and assembled using HiFiASM [35].

HiC libraries are constructed using the Arima Genomics HiC 2.0 kit coupled with the Swift Biosciences Accel-NGS 2S Plus kit for final library preparation. The final library is quantified by qPCR and sequenced on an Illumina platform, collecting 2 × 150 bp paired-end reads on an Illumina platform (Illumina, San Diego, CA, USA). If the HiC data is from the same individual as the HiFi reads, the former may be included as part of the HiFiASM input to allow for further haplotype resolution and phasing during assembly (Figure 1). This inclusion of HiC data increases contig resolution by HiFiASM beyond what can be achieved using HiFi reads alone [35]. Regardless of whether the HiC library was constructed from the same or different specimen, the HiC reads are used to build a proximity matrix (i.e., contact map) [36] for scaffolding using automated or semi-automated methods [37]. Manual editing is performed using the Juicebox Assembly Toolkit to produce highly accurate scaffolds that encompass entire chromosomes in some cases [38].

### 2.4. Mitochondrial and Contaminant Screening

Mitochondrial contigs are identified in each assembled genome using the MitoHiFi pipeline [39]. MitoHiFi implements a BLAST search for contigs that have a high similarity to whole mitochondrial genome sequences from the same or closely related species [40], selecting the contig with the greatest similarity and checking for circularization. Mitochondrial genes are then structurally annotated using intervals from the same mitochondrial genome used in the BLAST search through the MitFi annotation program in the MitoFinder pipeline [41,42]. The results from these analyses include a complete assembled mitochondrial genome (Figure 1) and a set of mitochondrial genome contigs that represents length polymorphisms in the non-coding and AT-rich mitochondrial control region that was difficult to sequence and assemble prior to the adoption of PacBio long-read sequencing technology.

Contigs that are likely microbial in origin are identified through the Blobtools2 [43] pipeline, wherein BLAST+ [40] and Diamond BLAST [44] are used to search for alignments of the assembled contigs against regularly updated nucleotide and reference protein databases, respectively. Alignment results are summarized using Blobtools2 to assign contigs to the taxon with the greatest cumulative bitscore. Unplaced contigs that are identified as Arthropoda are retained along with those not receiving a database “hit” or those that are undefined. All other contigs are removed from the assembly on the condition that they may represent environmental or wet-lab contamination.

Concurrent with the BLAST+ and Diamond BLAST searches, hierarchical BUSCO v3 [45,46] is used to assess an assembly for completeness. The BUSCO “genome” mode (-m genome’) implements AUGUSTUS [47], the “tBLASTn” function of BLAST+ [40], and HMMER [48] to detect the presence and completeness of single-copy orthologous genes in Eukaryota, Metazoa, Arthropoda, and Insecta databases. If necessary, Hemiptera, Endopterygota, Hymenoptera, and Diptera ortholog databases may be used. Results from the lowest taxonomic rank are reported, and unplaced contigs that contain BUSCOs that are duplicated on larger scaffolds are removed from the assembly.

### 2.5. Genome Annotation

#### 2.5.1. Structural and Functional Annotation

Structural annotation refers to the prediction of gene structures on a genome assembly, including the positions of transcripts, exons, introns, coding sequences, and other features [49]. Functional annotation provides information about the gene’s biological role(s), for example, gene ontologies [50], pathways, functional domains, and names. Model organism databases can manually assign biological function to genes by accumulating evidence from the scientific literature and structuring it in human and machine-readable formats. In contrast, for non-model organisms such as those in the Ag100Pest Initiative, most, if not all, functional annotation is performed computationally, as (1) gene function in very few genes have been established experimentally for these non-model species, and (2) the capacity for literature-based curation of gene function does not yet exist for these species.

Most of the genome assemblies generated by the Ag100Pest project are being annotated using the NCBI eukaryotic annotation pipeline [51]. This pipeline relies on Gnomon [52] for gene prediction and uses genome assembly, RNA sequencing (RNA-Seq) alignments, transcripts, and protein alignments as inputs. The resulting gene predictions are given an accession number and made publicly available. Gene names are assigned based on homology to proteins in SwissProt [53,54]. The NCBI eukaryotic annotation pipeline requires both the genome assembly and associated RNA-Seq evidence to be publicly available in the NCBI’s GenBank and Sequence Read Archive, respectively (SRA; see [55]). In the event that an Ag100Pest species lacks sufficient RNA-Seq evidence in SRA, additional data will be generated, as appropriate, and submitted to aid with NCBI gene structure prediction and annotation.

NCBI does not currently generate additional functional annotations. Proteins deposited in GenBank or generated by RefSeq should eventually be functionally annotated by UniProt [53]. To provide immediate and consistent functional annotation of RefSeq models from genomes assembled by the Ag100Pest Initiative, a functional annotation workflow for arthropod genomes was developed [56], described in a separate paper in this special issue. This pipeline uses GOAnna [57] and InterProScan [58] for Gene Ontology [50] (GO) and protein domain annotation and KOBAS [59] for annotation with KEGG (Kyoto Encyclopedia of Genes and Genomes) pathways [60]. The i5k Workspace@NAL platform [61] will compute and provide access to these functional annotations until they are superseded by functional annotations from UniProt, after which they will be archived.

#### 2.5.2. Manual Annotation

Automated structural and functional annotations can rapidly provide information on gene models and their putative biological roles. However, these predictions are not always correct due to many factors, including problematic genome assemblies or rapidly evolving gene families and paralogous genes in tandem arrays that are difficult to predict using structural annotation programs. In these cases, models must be manually reviewed and updated. The Ag100Pest project supports the manual improvement of RefSeq’s gene predictions via manual curation tools at the i5k Workspace@NAL platform [61], including Apollo software [62] and mapped RNA-Seq to validate gene structures. Manual improvements of these gene predictions are vetted and submitted back to NCBI GenBank, where they can be used as transcript or protein alignments to improve future gene predictions. 

### 2.6. Data Management

Ag100Pest data is intended as a resource and infrastructure to be used by the larger scientific community. Thus, proper data management is a cornerstone of Ag100Pest project design. Genome projects generate several data types, all with associated metadata that describe what the data are and where they came from. Our goal is not only to follow community best practices for the data types generated during a genome project but also to provide as rich and consistent metadata as possible to maximize the potential for re-use of the data. Data types, their metadata, and final repositories are listed in the supplementary materials (Table S1). All data are deposited at NCBI’s databases, which are the community-accepted primary archives for nucleotide and protein data and metadata.

We created an umbrella NCBI BioProject for all Ag100Pest submissions [63]. All data associated with the Ag100Pest project will be available under this accession number. Metadata associated with each project was collected during the sample submission process via custom submission templates. All projects used the Invertebrate 1.0 BioSample package [64] for sample metadata in order to streamline metadata collection and later search and retrieval. Primary archiving of these datasets at NCBI is critical for community re-use. In addition, we are making the data available through the insect community database at the i5k Workspace@NAL platform [61] for further interaction and updates. The i5k Workspace@NAL platform will provide additional functional annotations (see above) as well as community annotation tools for manual annotation and refinement of gene predictions and other community database services. As such, the Ag100Pest initiative provides end-to-end genome project data management, delivering database access and associated tools to the research community in addition to the data and genome assemblies.

## 3. Results

**Figure 2:**
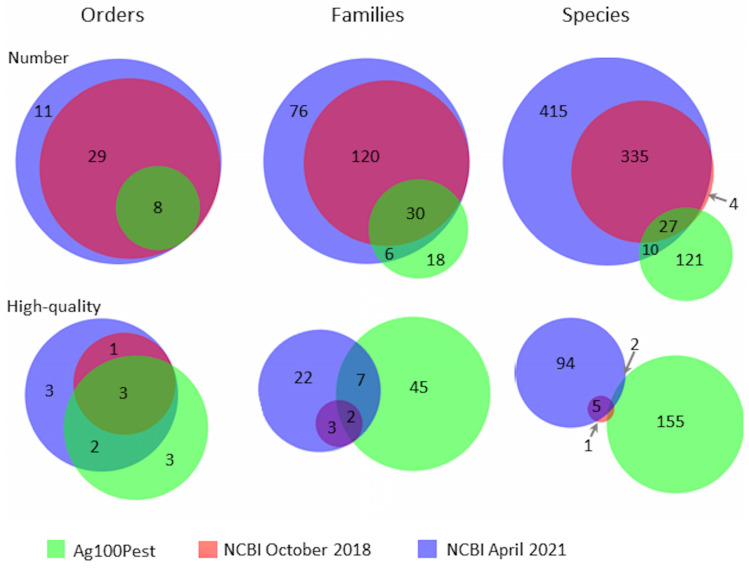
Venn diagrams showing the number of species present in NCBI for the phylum Arthropoda at the initiation of the Ag100Pest Initiative and at present compared to the species included in the Ag100Pest Initiative. NCBI data was accessed on 24 October 2018 and 27 April 2021, respectively. The top row includes all species present in NCBI, and the bottom row includes only those species with an assembly deemed high-quality at the taxonomic levels of order, family, and species. Assemblies with a contig N50 of 1 Mbp or greater and scaffolding with an N50 of 10 Mbp or greater were deemed high-quality. Assemblies without clear scaffolding (scaffold N50 > contig N50) were not evaluated as high-quality. We strive to produce high-quality genome assemblies for all species covered by the Ag100Pest Initiative.

**Figure 3:**
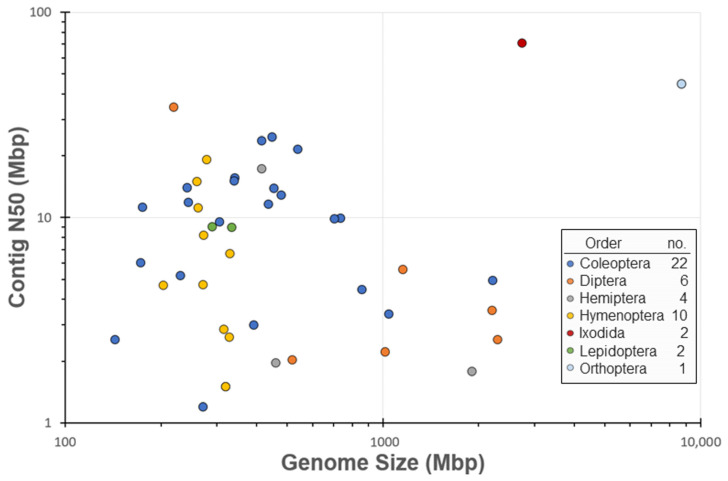
Plot of contig N50 (Mbp) versus genome size (Mbp) for Ag100Pest assemblies. This data was gathered from 47 insect genomes that have completed HiFi sequencing and were assembled by the Ag100Pest Initiative. Assemblies that require additional sequencing to achieve a high-quality assembly were excluded from the dataset. The data was plotted on a logarithmic axis to reduce skew from outliers, and data points were color-coded based on order.

The Ag100Pest Initiative has prioritized the sequencing and assembly of genomes from 158 species from 54 families across 8 arthropod orders. This includes 18 families and 121 species that lack a publicly available assembly of any quality (Figure 2; species list at [27]). The total number of assemblies in progress will be higher than the number of species as we are sequencing multiple isolates, biotypes, subspecies, or sexes for some species. Selection of species for the Ag100Pest Initiative was made on the basis of their status as important beneficial or pest species, as opposed to maximizing taxonomic breadth. Nevertheless, we will make a substantial contribution to the EBP goal of generating a reference assembly for a representative of every eukaryotic family and an assembly for every species [1]. Toward this end, our focus on high-quality assemblies (defined, in part, by the Vertebrate Genomes Project (VGP) [65] as contiguity measures of contig N50 > 1 Mbp and scaffold N50 > 10 Mbp) will elevate the overall contiguity and accuracy of arthropod genomes in the public domain and provide a family level representative for 45 families across 3 orders that currently lack a high-quality assembly for any species (Figure 2). A notable impact in the order Coleoptera is expected with our goal of contributing 50 assemblies, nearly doubling the current number of 54 lower-quality coleopteran public assemblies (Table 1). The contig assemblies already generated for almost half of the intended Ag100Pest coleopteran genome assemblies (22 species; Figure 3) surpass the contig contiguity of the majority of publicly released assemblies for this order. Other similarly substantial impacts will be made for orders Hemiptera, Hymenoptera, Ixodida, and Orthoptera (Table 1).

Ag100Pest began by using continuous long reads (CLRs) for assembly (details not presented herein) as the improved HiFi procedure [33] had not yet been developed. Working in collaboration with Pacific Biosciences, methods for low DNA input library preparation and HiFi sequence generation were developed that were key to the success of the Initiative. The choice of library preparation method is highly dependent on individual samples and beyond the scope of this project overview. However, key aspects for consideration are organism size (i.e., the amount of DNA available for an individual sample), difficulty of extraction (i.e., the quality and size distribution of DNA fragments), and genome size. The methods available range from ultra-low input methods, suitable when the genome size is less than 1 Gbp and the specimen size is very small, to standard library preparation methods when the individuals are relatively large and the genome size is also large and requires multiple sequencing runs to achieve desired coverage. For most insects, we find the low-input protocol [66] is the best compromise between the three available library preparation methods as we find that it performs well for relatively small insects over a range of genome sizes.

The majority of selected Ag100Pest species do not have existing public assemblies; however, 37 species with relatively low-quality assemblies were included to improve their assembly quality (Figure 2). We have generated contig-level assemblies for 11 of these 37 (Table 2), 10 of which we improved contig N50 by several orders of magnitude. The exception, *Haemaphysalis longicornis*, illustrates the difficulties inherent in a project attempting to assemble a broad diversity of Arthropoda genomes. Our initial contig N50 showed only a modest improvement over the previous assembly. Likely because *H. longicornis* present in the United States appears to be parthenogenetic and is, therefore, either triploid or aneuploid [67], our assembly size is substantially larger than the predicted genome size. This suggests the presence of haplotypic duplication that complicates the generation of a single haplotype representation of a polyploid genome [35]. We anticipate that the contig N50 of our assembly will improve after the haplotypic duplication is removed [68] because the alternate haplotype contigs tend to be smaller and, therefore, artifactually reduce the N50 value. Nevertheless, this species illustrates one example of the challenges inherent in developing a “one-size-fits-all” pipeline applied to the huge diversity of arthropod species.

For the 47 species distributed across seven orders for which we have completed HiFi long-read sequencing and contig assemblies, our assembly lengths range from 144 to 8.7 Gbp, with contig N50s ranging from 0.88 to 70 Mbp (Figure 3, Table S2). Final contig N50 and assembly sizes for these assemblies may change during the scaffolding and contamination removal steps. After the completion of these processes, the assemblies will be deposited into NCBI. The Ag100Pest initiative is committed to the free and open access of all data in the public domain while still maintaining defined ownership of input specimens and assembly outputs through academic research agreements to protect the interests of all parties involved.

## 4. Discussion

The Ag100Pest Initiative was launched in October 2018, at which time only 6 of 366 (1.6%) arthropod genomes then available through NCBI met our standards of contiguity (taken from those [65] of the Vertebrate Genomes Project (VGP) for defining high-quality assemblies). Therefore, while producing genome assemblies that met the VGP standard was possible at the time for a handful of species, it was not straightforward for the majority of arthropods due to technological and biological issues. Ag100Pest’s goal to produce reference-quality assemblies was, therefore, all the more audacious in 2018 because we intended to sequence at scale, with long-read sequencing coming from a single specimen, not pools, for a wide variety of species across several taxa. The success of our project has not only allowed it to expand beyond the initial intended 100 species but to provide a framework by which other initiatives can also contribute to the lofty goal of the EBP to sequence all known eukaryotic species.

The inability to produce long-read data from single specimens was a technological challenge that hindered assemblies in the past, fracturing assemblies and inflating the number of haplotigs that originated from the same genomic interval. Advances in genomic DNA isolation, long-read library construction, and sequencing [69] have been fundamental to the success of the Ag100Pest Initiative, helping to ensure the assemblies produced by Ag100Pest will meet or exceed quality metrics established by the EBP [26] and VGP [65]. Our continuous integration and refinement of new methods to address particular challenges posed by arthropods have allowed Ag100Pest to sequence species that were not tractable when we began this project. Specifically, the reduction in input DNA requirements since the project’s inception has generated low and ultra-low input protocols for long-read sequencing libraries [66,70] that have allowed us to sequence species with very small physical sizes. Additionally, PacBio’s optimization of circular consensus sequencing (CCS) greatly increased the sequencing accuracy and generation of High-Fidelity (HiFi) reads [33], which hold many benefits over CLR. With these decreases in input requirements and increases in output accuracy, sequencing data can be generated from a single specimen rather than pools of specimens. Assembly phasing is, therefore, improved and the introduction of additional heterozygosity into the assembly graph is reduced, resulting in a more complete and contiguous assembly. Long-read sequencing technology now enables high-quality arthropod genome sequencing and assembly across the broad diversity of arthropods.

Unfortunately, some species still present unique challenges to DNA extraction, sequencing efficiency, and assembly contiguity, and, often, these cannot be anticipated in advance. We have found that sequencing output varies across species and cannot always be attributed to sample quality. In general, we found that sequencing success was most improved when HiFi libraries were immediately prepared from recently extracted DNA that had not been frozen, stored for long periods of time, or shipped. Therefore, we do not recommend shipping extracted high molecular weight (HMW) DNA to a sequencing facility for library preparation and sequencing. Instead, we recommend either sending the specimen itself to the facility for DNA extraction and library preparation or preparing libraries before shipping. Additionally, while highly accurate CCS long-read sequencing that produces HiFi reads is currently the best approach to resolving repetitive genome architecture, regions with large arrays of highly similar repeats, longer than the sequencing reads themselves, may remain difficult to assemble without the incorporation of ultra-long reads. These remaining challenges are small in comparison to the state of the field just two years ago, when only a small fraction of assemblies met high-quality standards (Figure 3).

Only 101 of 787 (12.8%) arthropod species currently have a genome assembly in the public domain that meets the definition of high-quality (Figure 2). With the advancements noted above, highly accurate, low-cost sequencing technology and genome assembly methods are no longer the limiting factors for producing high-quality genome assemblies in the vast majority of arthropods despite the wide range of physical and genome size challenges they present. By adopting the latest sequencing and assembly methods and paying particular attention to details such as proper specimen preservation, reference genome assemblies can be produced by all sequencing consortia. We encourage other sequencing consortia to commit to the production of high-quality genome assemblies in order to advance both the phylogenetic breadth of sequenced species and their overall contiguity and completeness.

## 5. Conclusions

The high-quality genome assemblies Ag100Pest is producing for pest arthropods are fundamental infrastructure for basic and applied research. One benefit of having the USDA-ARS undertake this project is that Ag100Pest can leverage personnel and infrastructure resources by making investments in permanently funded staff, sequencing platforms, and computational support that are not limited by typical granting cycles. USDA-ARS scientists also possess unique expertise in arthropod pest management and agricultural genomics research across a wide breadth of commodities and cropping systems. Sequencing of arthropods advances our understanding of the physiology, ecology, and evolution of pests and beneficial arthropods. Translational research products based on that knowledge will lead to improvements in the agricultural economy that will come to agricultural producers through technological advances in the efficacy and durability of environmentally sustainable pest management practices. For example, high-quality genome assemblies are used in the development of novel molecular-based management tools that target pests while sparing environmental damage, particularly damage to beneficial arthropod populations. As such, the accumulation of genome assemblies for arthropods contributes to a foundation of support for the bioeconomy. Increasing profitability while reducing any negative environmental impacts of agricultural production directly benefits rural economies, societal well-being, and overall human health. Maintaining the quantity, quality, and stability of production is critical to global food security that is required to provide nutritious food to a growing human population as well as raw materials for industrial production of bio-based products. The Ag100Pest Initiative addresses this multitude of stakeholder needs through the development of high-quality foundational genomic information that is anticipated to facilitate the development of novel tools and products for the targeted management of pests and the preservation of beneficial insect health. While these and other outcomes, as well as changing stakeholder needs, will continue to reprioritize objectives within the Ag100Pest Initiative, we remain committed to supporting the scientific community and agricultural and societal interests.

## Figures and Tables

**Table 1:** Number of species with a genome assembly in NCBI or included in the Ag100Pest Initiative for eight orders in the phylum Arthropoda, covered by the Ag100Pest Initiative. NCBI data was accessed on 24 October 2018 and 27 April 2021, respectively. The number of species with a high-quality assembly in NCBI for each order is indicated in parentheses. Assemblies with a contig N50 of 1 Mbp or greater and scaffolding with an N50 of 10 Mbp or greater were deemed high-quality. Assemblies without clear scaffolding (scaffold N50 > contig N50) were not evaluated as high-quality.

Order	NCBI Oct 2018	NCBI Apr 2021	Ag100Pest
Coleoptera	16 (0)	54 (0)	50
Diptera	119 (3)	186 (31)	25
Hemiptera	27 (0)	51 (4)	37
Hymenoptera	73 (1)	169 (6)	15
Ixodida	3 (0)	11 (1)	10
Lepidoptera	53 (1)	149 (52)	16
Orthoptera	3 (0)	5 (0)	4
Thysanoptera	1 (0)	3 (0)	1

**Table 2:** Improvement of Ag100Pest contig assemblies over publicly available assemblies. NCBI data was accessed on 27 April 2021.

Order	Family	Scientific Name	TaxID	Common Name	NCBI Representative Assembly	NCBI Assembly Date	NCBI Assembly Length (Mbp)	NCBI Contig N50 (Mbp)	Ag100Pest Assembly Length (Mbp)	Ag100Pest Contig N50 (Mbp)
Coleoptera	Silvanidae	*Oryzaephilus surinamensis*	41112	saw-toothed grain beetle	GCA_004796505.1	16 April 2019	104.01	0.019	173.49	5.98
Coleoptera	Tenebrionidae	*Tribolium castaneum*	7070	red flour beetle	GCF_000002335.3	10 March 2016	165.94	0.073	242.40	13.86
Diptera	Muscidae	*Stomoxys calcitrans*	35570	stable fly; biting house fly	GCF_001015335.1	31 May 2015	971.19	0.011	1159.87	5.56
Hemiptera	Aphididae	*Aphis gossypii*	80765	cotton aphid; melon aphid	GCF_004010815.1	10 January 2019	294.28	0.077	416.81	17.16
Hymenoptera	Diprionidae	*Neodiprion lecontei*	441921	redheaded pine sawfly	GCA_001263575.2	21 June 2018	239.78	0.087	273.27	8.16
Hymenoptera	Diprionidae	*Neodiprion pinetum*	441929	white pine sawfly	GCA_004916985.1	26 April 2019	269.78	0.016	272.19	4.68
Hymenoptera	Formicidae	*Wasmannia auropunctata*	64793	little fire ant	GCF_000956235.1	17 March 2015	324.12	0.038	320.50	1.49
Hymenoptera	Vespidae	*Vespula pensylvanica*	30213	western yellowjacket	GCA_014466175.1	9 September 2020	179.37	0.097	204.70	4.64
Ixodida	Ixodidae	*Haemaphysalis longicornis*	44386	longhorned tick	GCA_013339765.1	16 June 2020	2554.97	0.740	5576.40	0.88
Lepidoptera	Pyralidae	*Plodia interpunctella*	58824	Indianmeal moth	GCA_900182495.1	6 May 2017	382.24	0.312	291.43	8.96

## Data Availability

Sequencing data and assemblies are made available through the NCBI Ag100Pest Umbrella BioProject: PRJNA555319 as well as through the i5k Workspace@NAL platform.

## Supplementary Materials

The following are available online at https://www.mdpi.com/article/10.3390/insects12070626/s1, Table S1: Data types generated by the Ag100Pest project and their repositories, Table S2: Ag100Pest assembly metrics.
