# Supplementary material for: The USDA-ARS Ag100Pest Initiative: High-Quality Genome Assemblies for Agricultural Pest Arthropod Research
Source: Insects. 2021 Jul 9;12(7):626. doi: 10.3390/insects12070626 (PMC8307976; doi:10.3390/insects12070626)
Supplement: Supplementary file 1 [file insects-12-00626-s001.zip › insects-1266526-supplementary.pdf]

insects MDPI

Table S1. Data types generated by the Ag100Pest project and their repositories.

Data Type	Repositories	Data submission notes
Project	NCBI BioProject (https://www.ncbi.nlm.nih.gov/bioproject), i5k Workspace@NAL (http://i5k.nal.usda.gov)	Ag100Pest Umbrella BioProject: PRJNA555319
Physical Sample	National repositories, for example GGBN/GBIF	It is our intention to provide vouchers and exemplars as available to the Smithsonian Global Genome Initiative (GGI, https://naturalhistory.si.edu/research/global-genome-initiative).
Sample metadata	NCBI BioSample (https://www.ncbi.nlm.nih.gov/biosample), i5k Workspace@NAL (https://i5k.nal.usda.gov/)	All Ag100Pest BioSamples use the Invertebrate 1.0 Sample package (https://www.ncbi.nlm.nih.gov/biosample/docs/packages/Invertebrate.1.0/)
DNA and RNA high-throughput sequencing data	NCBI SRA (http://www.ncbi.nlm.nih.gov/sra)	NA
Genome assembly	NCBI assembly (https://www.ncbi.nlm.nih.gov/assembly/), i5k Workspace@NAL	NA
Gene structural annotations	NCBI RefSeq (https://www.ncbi.nlm.nih.gov/refseq/), i5k Workspace@NAL (https://i5k.nal.usda.gov/)	The NCBI RefSeq eukaryotic annotation pipeline generates gene structural annotations.
Protein functional annotations	i5k Workspace@NAL (https://i5k.nal.usda.gov/)	The AgBase functional annotation workflow is used to generate protein functional annotations.
Gene manual annotations	i5k Workspace@NAL (https://i5k.nal.usda.gov/), NCBI GenBank (https://www.ncbi.nlm.nih.gov/genbank/)	Community curators at the i5k Workspace@NAL using the Apollo software perform gene manual annotations.

Table S2. Ag100Pest assembly metrics. Table of assembly size and contig N50 for data presented in Figure 3. Data includes 47 species over 7 orders for which Ag100Pest has completed HiFi sequencing and contig assemblies.

Species	Order	Assembly Length (Mbp)	Contig N50 (Mbp)
Anthonomus grandis grandis	Coleoptera	862.04	4.42
Anthonomus grandis thurberi	Coleoptera	738.84	9.89
Aphthona nigriscutis	Coleoptera	1,048.75	3.38
Curculio caryae	Coleoptera	2,223.01	4.92
Cylas formicarius	Coleoptera	394.20	2.97
Cynaeus angustus	Coleoptera	244.95	11.79
Diabrotica undecimpunctata	Coleoptera	540.93	21.43
Diorhabda carinulata	Coleoptera	417.24	23.46
Diorhabda carinata	Coleoptera	448.66	24.53
Diorhabda elongata	Coleoptera	481.40	12.82
Diorhabda sublineata	Coleoptera	456.34	13.82
Gnatocerus cornutus	Coleoptera	342.84	15.45
Lasioderma serricorne	Coleoptera	231.76	5.19
Latheticus oryzae	Coleoptera	175.49	11.19
Oryzaephilus mercator	Coleoptera	144.03	2.52
Oryzaephilus surinamensis	Coleoptera	173.49	5.98
Trogoderma granarium	Coleoptera	307.73	9.49
Tribolium brevicornis	Coleoptera	706.82	9.82
Tribolium castaneum GA-2	Coleoptera	242.40	13.86
Tribolium castaneum Wakefield	Coleoptera	272.48	1.19
Tribolium confusum	Coleoptera	438.46	11.54
Trogoderma variabile	Coleoptera	342.38	15.00
Chaetopsis massyla	Diptera	220.32	34.36
Euxesta stigmatias	Diptera	521.07	2.02
Musca autumnalis female	Diptera	2,306.19	2.53
Musca autumnalis male	Diptera	2,210.83	3.52
Stomoxys calcitrans	Diptera	1,159.87	5.56
Strauzia longitudinalis	Diptera	1,019.91	2.20
Aphis gossypii	Hemiptera	416.81	17.16
Anasa tristis	Hemiptera	1,917.22	1.77
Bactericera maculipennis	Hemiptera	462.30	1.95
Mealy Bug	Hemiptera	320.50	1.49
Diprion similis	Hymenoptera	280.20	19.00
Neodiprion lecontei	Hymenoptera	273.27	8.16
Neodiprion pinetum	Hymenoptera	272.19	4.68
Neodiprion virginianus	Hymenoptera	263.17	11.04
Neodiprion fabricii	Hymenoptera	260.84	14.83
Vespa crabro	Hymenoptera	331.76	6.61
Vespa mandarina female	Hymenoptera	329.49	2.59
Vespa mandarina male	Hymenoptera	317.48	2.84
Vespula pensylvatica	Hymenoptera	204.70	4.64
Wasmannia auropunctata	Hymenoptera	320.50	1.49
Dermacentor andersoni	Ixodida	2,752.49	70.43
Haemaphysalis longicornis	Ixodida	5,576.40	0.88
Plodia interpunctella	Lepidoptera	291.43	8.96
Sitotroga cerealella	Lepidoptera	334.95	8.92
Schistocerca gregaria	Orthoptera	8,746.28	44.58
